# Cognitive mechanisms and resilience in UK-based general practitioners: cross-sectional findings

**DOI:** 10.1093/occmed/kqad016

**Published:** 2023-02-06

**Authors:** F O Kaleta, C B Kristensen, M Duncan, P Crutchley, P Kerr, C R Hirsch

**Affiliations:** King’s College London, Department of Psychology, Institute of Psychiatry, Psychology and Neuroscience, London SE5 4AF, UK; King’s College London, Department of Psychology, Institute of Psychiatry, Psychology and Neuroscience, London SE5 4AF, UK; King’s College London, Department of Psychology, Institute of Psychiatry, Psychology and Neuroscience, London SE5 4AF, UK; Harrow Community Mental Health Team, Central and North West London NHS Foundation Trust (CNWL), Bentley House, Harrow HA3 5QX, UK; Cornerways Surgery, Bromley BR3 5LG, UK; King’s College London, Department of Psychology, Institute of Psychiatry, Psychology and Neuroscience, London SE5 4AF, UK; South London and Maudsley NHS Foundation Trust, Bethlem Royal Hospital, Monks Orchard Road, Beckenham, Kent BR3 3BX, UK

## Abstract

**Background:**

Being a general practitioner (GP) is a stressful occupation, and the strain GPs are under can have negative effects on their psychological well-being, as well as on the patients’ experience of healthcare. Resilience can help buffer against this and is a dynamic process by which one can cope with adversity and stress.

**Aims:**

This study aimed to identify modifiable cognitive mechanisms related to resilience in GPs, specifically interpretation bias and cognitive reappraisal.

**Methods:**

One hundred and fourteen GPs completed an online cross-sectional correlational study. This comprised questionnaires assessing resilience, emotional distress, work environment and cognitive mechanisms (emotion regulation), as well as a task assessing interpretation bias.

**Results:**

Resilience of GPs was negatively correlated with measures of emotional distress. Furthermore, resilience was positively correlated with positive interpretation bias (*r* = 0.60, ρ = 0.60, *P* < 0.01) and cognitive reappraisal (*r* = 0.39, ρ = 0.40, *P* < 0.01). In a hierarchical regression, positive interpretation bias (*B* = 0.25, SE *B* = 0.06, β = 0.39, *P* < 0.01) was a significant independent predictor of resilience when controlling for depression, anxiety and stress.

**Conclusions:**

This is the first study to establish an association between resilience and positive interpretation bias and cognitive reappraisal in GPs. Future research should use longitudinal designs to determine if they have a causal role in promoting resilience, and importantly whether interventions focusing on these processes may foster resilience in less resilient GPs.

## Teaser

General practitioners (GPs) work under long-term stress. Resilience reduces the impact of stress, including its negative effects on well-being. Two modifiable psychological mechanisms, tendencies to make positive interpretations and helpful cognitive reappraisals, were studied cross-sectionally in relation to GPs’ resilience. The study established, for the first time, an association between higher levels of both positive interpretations and helpful cognitive reappraisals, and greater resilience in GPs.

**Key learning points**

**What is already known about this subject:**

- GPs need to be resilient in the face of high occupational stress.
- High occupational stress can lead to negative outcomes for the GP and for the patient.
- No research has assessed what cognitive mechanisms are associated with resilience in UK-based GPs.

**What this study adds:**

- Our study identified that interpretation bias, emotion regulation and psychosocial work environment are associated with greater resilience in UK-based GPs.

**What impact this may have on practice or policy:**

- Findings may help to develop interventions that target modifiable cognitive mechanisms to promote resilience in occupations that experience high stress, making our research translational in nature.
- Our research contributes important findings to the limited literature on resilience in UK-based GPs and primary care settings.

## Introduction

A recent report found that UK-based general practitioners (GPs) are among the most stressed and least satisfied in the profession when compared to their international colleagues, citing high workload, short consultation time and unsatisfactory pay [1]. High occupational stress poses potential risks to the patient and the GP. For the patient, it may lead to lower accessibility and availability of care [2], and for the GP it may lead to depression, anxiety and burnout [3].

Resilience is a dynamic process that enables a person to adapt and recover from stress and adversity and has consistently been linked with psychological well-being in other healthcare professionals [4–6]. Examination of the link between resilience and psychological well-being in UK-based GPs is vital given the substantial prevalence of occupational stress.

Interpretation bias is the tendency to draw positive or negative conclusions from ambiguous and/or uncertain situations. A negative interpretation bias has been associated with individuals experiencing high levels of burnout and depression [7,8]. Positive interpretation bias is a key cognitive mechanism in predicting resilience in women living beyond cancer [9] and in unpaid caregivers [10]. Moreover, computerized training of positive interpretations reduced worry, anxiety and depression in people with generalized anxiety disorder and depression [11], suggesting that interpretation bias is an important mechanism in promoting well-being. The current study will examine whether resilience is associated with more positive interpretations in GPs.

Cognitive reappraisal is associated with resilience. This has recently been demonstrated in a sample of adolescents during the COVID-19 pandemic [12]. There is limited research on the relationship between resilience and another form of emotion regulation, namely expressive suppression. However, some studies have demonstrated that expressive suppression is associated with long-term adverse effects on well-being [13]. The current study will examine the relationship between levels of resilience and cognitive reappraisal and expressive suppression in GPs, enabling us to identify mechanisms that are likely to promote resilience.

Recent research indicates that system and organizational factors, including work intensity, are detrimentally impacting GP well-being and retention [14]. For example, the 2021 ‘Eleventh National GP Worklife Survey’ indicated that over 80% of GPs report high/considerable pressure from increased patient demands and workload, with a third of GPs reporting a considerable/high likelihood of leaving the profession within the next 5 years [15]. Whilst helpful policy and practice changes to the nature of GP work and workload have been suggested [14], it is evident that there may be some delay to implementation of system changes. Given this, it is vital to look at wider ways in which the high levels of occupational stress experienced by GPs may be supported. One way in which to do this may be via identifying and exploring modifiable cognitive mechanisms that may promote resilience.

To date, no research has been conducted on the cognitive mechanisms of resilience in UK-based GPs. It is imperative to demonstrate the significance of these cognitive mechanisms in GPs, to support the development of effective, accessible psychological interventions that promote resilience in GPs. We conducted a cross-sectional, online study with self-report questionnaires and an interpretation bias task to examine the relationship between resilience and the following factors: negative emotional symptoms, interpretation bias, cognitive reappraisal, expressive suppression and psychosocial work environment; in a sample of UK-based GPs.

## Methods

Participants were recruited through digital advertisements, e-mail bulletins and social media between January and August 2021. Ethical approval was granted by the Psychiatry, Nursing and Midwifery Research Ethics Subcommittees at King’s College London (HR-19/20-20950). To take part, participants had to be based in the UK, be below the age of 70 years and be working primarily as a GP in the past year. Participants were excluded pre-analyses if they completed less than 80% of the study and if they unscrambled 50% or more of the sentences in the Scrambled Sentences Test (SST) incorrectly.

A statistical power analysis was based on data from previous research exploring stress in England-based teachers [16]. The effect size (ES) in this study was *R*^2^ 0.32, with an alpha = 0.05 and power = 0.80. Thus, the projected sample size needed with this ES was approximately *n* = 74. Due to greater diversity between GP roles, the intended sample was extended to *N* = 100, but we were fortunate to over-recruit.

Data collection was conducted online and was incentivized with a prize draw. First, participants provided consent and answered demographic questions. Next, participants completed a series of questionnaires and a cognitive task, all presented in a randomized order. Questionnaires included the 25-item Connor–Davidson Resilience Scale (CDRS; Cronbach’s α in this study = 0.92; McDonald’s ω = 0.92 [17]) to assess resilience, and the Depression, Anxiety, and Stress Scale (DASS-21 [18]) was used to assess negative emotional symptoms (α = 0.95; ω = 0.95), and its subscales were used to assess depression (α = 0.92; ω = 0.92), anxiety (α = 0.87; ω = 0.88) as well as stress (α = 0.87; ω = 0.87).

The Managements Standards Indicator Tool (MSIT [19]) was administered to measure the psychosocial work environment, which includes factors such as perceived workload, staffing and demand placed upon the employee, and informs us about the levels of occupational stressors experienced by the studied sample (α = 0.94; ω = 0.94). The Emotion Regulation Questionnaire (ERQ [20]) was administered to measure two cognitive constructs captured by its subscales, namely cognitive reappraisal (α = 0.86; ω = 0.86), and expressive suppression (α = 0.85; ω = 0.88). The third cognitive mechanism, interpretation bias, was assessed through the 10-item SST (based on [21]). The 10 items used for this shortened version of the SST were developed to capture worry-related interpretations [11]. For each sentence, participants were presented with six randomly ordered words and were asked to select five of these words in the correct order to make a grammatically correct sentence. Each sentence had two viable versions, one emotionally positive, and one emotionally negative. Participants had two and a half minutes to unscramble as many sentences as possible. The time frame was based on a previous study where participants had 5 min to unscramble 20 sentences (double the amount in the present study [22]). The internal consistency of the SST was moderate (α = 0.52, Guttman split-half coefficient = 0.48). Following completion of the questionnaires and cognitive task, participants then completed further demographic questions (see appendices for demographic questions). To process the raw data, 12 negatively worded items on the MSIT were reverse-coded, whereby a score of 1 was turned into 5, a score of 2 into 4 and so on. DASS-21 scores were grouped into one total distress score, as well as three dimension scores (depression, anxiety and stress). The ERQ scores were grouped into subscale totals (cognitive reappraisal and expressive suppression). The SST positive interpretation bias score was computed as the proportion of positively unscrambled sentences out of the total number of grammatically correct sentences unscrambled.

The subsequent statistical analyses were conducted in IBM SPSS Statistics (version 27.0). The descriptive statistics of the continuous variables were reported with means and standard deviations. Demographic categorical variables were reported using frequencies and percentages. Correlational analyses were conducted between resilience and the following variables: negative emotional symptoms; psychosocial work environment, interpretation bias and emotion regulation (cognitive reappraisal and expressive suppression).

Hierarchical regression was used to conduct further analyses between resilience, negative emotional symptoms, interpretation bias and cognitive reappraisal. In Step 1, resilience was regressed on depression, anxiety and stress. In Step 2, cognitive mechanisms, positive interpretation bias and cognitive reappraisal were added to the model. This allowed for an assessment of whether the cognitive mechanisms explained a unique variance in resilience, whilst controlling for negative emotional symptoms. This was done because negative emotional symptoms, such as anxiety or depressive symptoms, are related to resilience as well as the cognitive mechanisms in question.

## Results

The demographic characteristics of the total sample of 114 GPs are provided in Table 1. The sample had a high proportion of female respondents (91%), with most respondents working in an urban GP surgery location (72%).

**Table 1.** Participants’ personal and occupational characteristics (*N =* 114)

Characteristics	
	Mean (standard deviation)
Age	41.84 (7.01)
Years qualified as a GP	10.94 (7.62)
GP sessions worked a week	5.55 (1.67)
	Reported in % and (*n*)
Gender	
Male	8 (9)
Female	91 (99)
Prefer not to say	1 (1)
Ethnicity	
Chinese	1 (1)
Indian	14 (15)
Mixed/multiple ethnic groups	4 (4)
Other	4 (4)
Prefer not to say	1 (1)
White	77 (84)
Sexual minority—lesbian, gay, bisexual & other groups	4 (5)
GP surgery location	
Rural	28 (30)
Urban	72 (76)
Additional clinical roles	37 (42)
Additional non-clinical roles	23 (26)
Type of primary role	
GP partner or principal	40 (46)
Salaried GP	49 (56)
Locum GP	9 (10)
Portfolio GP	1 (1)
Other	1 (1)
Work as locum in addition to main role	14 (16)
Unpaid caregiver—including young children	47 (51)

Descriptive statistics for the questionnaire measures and the SST (from which the interpretation bias score was derived) are presented in Table 2.

**Table 2.** Descriptive statistics of the variables

Outcome measures	Mean (standard deviation)
CDRS (*N* = 113)	87.22 (13.61)
DASS-21	
Negative emotional symptoms—total (*N* = 111)	16.68 (12.46)
Depression (*N* = 111)	5.42 (4.96)
Anxiety (*N* = 111)	3.34 (3.95)
Stress (*N* = 111)	7.92 (4.66)
Positive interpretation bias score (*N* = 114)	0.68 (0.21)
Cognitive reappraisal—ERQ (*N* = 112)	27.73 (5.94)
Expressive suppression—ERQ (*N* = 112)	14.30 (5.60)
MSIT (*N* = 111)	111.00 (20.19)

We found a significant positive correlation between resilience and positive psychosocial work conditions, and significant negative correlations between resilience and general negative emotional symptoms, depression, anxiety, as well as stress (see Table 3). These negative correlations were significant at *P* < 0.01 and of moderate ES. The exploratory analysis yielded a significant negative correlation between resilience and emotion regulation mechanism of suppressive expression.

**Table 3.** Correlations matrix of the measures

Correlations (*r,* ρ)	CDRS	DASS-21	DASS-21-D	DASS-21-A	DASS-21-S	MSIT	Positive interpretation bias	Cognitive reappraisal— ERQ	Expressive suppression—ERQ
CDRS (*n* = 113)	–	−0.47**, −0.50**	−0.49**, −0.52**	−0.39**, −0.40**	−0.39**, −0.40**	0.33**, 0.31**	0.60**, 0.60**	0.39**, 0.40**	−0.12, −0.16
DASS-21 (*n* = 111)	−0.47**, −0.50**	–	0.93**, 0.88**	0.90**, 0.80**	0.92**, 0.92**	−0.37*, −0.42**	−0.52**, −0.51**	−0.32**, −0.32**	0.20*, 0.19*
DASS-21-D (*n* = 111)	−0.49**, −0.52**	0.93**, 0.88**	–	0.74**, 0.58**	0.77**, 0.70**	−0.41**, −0.45**	−0.54**, −0.54**	−0.39**, −0.40**	0.16, 0.18
DASS-21-A (*n* = 111)	−0.39**, −0.40**	0.90**, 0.80**	0.74**, 0.58**	–	0.74**, 0.68**	−0.25**, −0.25**	−0.42**, −0.39**	−0.16, −0.18	0.26*, 0.29**
DASS-21-S (*n* = 111)	−0.39**, −0.40**	0.92**, 0.92**	0.77**, 0.70**	0.74**, 0.68**	–	−0.38**, −0.35**	−0.46**, −0.42**	−0.31**, −0.28**	0.15, 0.13
MSIT (*n* = 111)	0.33**, 0.31**	−0.39**, −0.42**	−0.41**, −0.45**	−0.25**, −0.25**	−0.38**, −0.35**	–	0.38**, 0.30**	0.26**, 0.28**	−0.09, −0.07
Positive interpretation bias score (*n* = 114)	0.60**, 0.60**	−0.52**, −0.51**	−0.54**, −0.54**	−0.42**, −0.39**	−0.46**, −0.42**	0.38**, 0.30**	–	0.43**, 0.43**	−0.31**, −0.33**
Cognitive reappraisal—ERQ (*n* = 112)	0.39**, 0.40**	−0.32**, −0.32**	−0.39**, −0.40**	−0.16, −0.18	−0.31**, −0.28**	0.26**, 0.28**	0.43**, 0.43**	–	0.01, −0.07
Expressive suppression—ERQ (*n* = 112)	−0.12, −0.16	0.20*, 0.19*	0.16, 0.18	0.26**, 0.29**	0.15, 0.13	−0.09, −0.07	−0.31**, −0.33**	0.01, −0.07	–

CDRS, Connor–Davidson Resilience Scale; DASS-21, Depression, Anxiety and Stress Scale total score; DASS-21-D, Depression subscale; DASS-21-A, Anxiety subscale; DASS-21-S, Stress subscale; MSIT, Management Standards Indicator Tool.

*P < 0.05, **P < 0.01.

Correlations between resilience and positive interpretation bias, as well as resilience and cognitive reappraisal, were positive, significant at *P* < 0.01 and of moderate ES.

When further investigating the relationships of resilience with interpretation bias and cognitive reappraisal, in Step 1 of the hierarchical regression resilience was regressed on DASS-21 subscales of depression, anxiety and stress (to control for their relationship with resilience) and the regression was significant (*F*(3, 105) = 11.38, *P* < 0.01, adjusted *R*^2^ = 0.22). In Step 2, positive interpretation bias and cognitive reappraisal, assessed using subscale of the ERQ, were added to the model. Their addition significantly explained a further 15% of the variance in resilience and this change in *R*² was significant (*F*(2, 103) = 12.94, *P* < 0.01, *R*^2^ change = 0.15). However, only positive interpretation bias was a significant independent predictor of resilience. See Table 4 for detailed statistics of the model.

**Table 4.** Statistics of the hierarchical regression model

	Adj *R*^2^	*B*	SE *B*	β	*P*
Step 1	0.22				<0.01
Constant		103.087	3.86		<0.01
Depression		-1.24	0.39	-0.46	<0.01
Anxiety		-0.16	0.46	-0.05	0.72
Stress		0.14	0.43	0.01	0.98
Step 2	0.37				<0.01
Constant		68.10	8.27		<0.01
Depression		-0.56	0.38	-0.21	0.14
Anxiety		-0.25	0.43	-0.08	0.55
Stress		0.14	0.39	0.05	0.72
Positive interpretation bias		0.25	0.06	0.39	<0.01
Cognitive reappraisal		0.32	0.21	0.14	0.12

## Discussion

This study was an online cross-sectional, correlational study that aimed to assess factors related to GPs’ resilience, in particular the cognitive mechanisms of interpretation bias and cognitive reappraisal. There were three key findings from this study. Firstly, GPs scored highly on both the resilience scale (*M* = 87.22) and on the scales of depression, anxiety and stress (*M* = 5.42; *M* = 3.32; *M* = 7.92). Secondly, resilience was found to be negatively correlated with negative emotions and was positively correlated with positive psychosocial work conditions. Thirdly, the cognitive mechanisms, positive interpretation bias and cognitive reappraisal had positive correlations with resilience, and positive interpretation bias was a significant independent predictor of resilience.

The resilience scores of GPs are higher than those in the general population (*M* = 80.4), primary care outpatients (*M* = 71.8) and in patients with generalized anxiety disorder (*M* = 62.4 [15]). Similarly, for depression, anxiety and stress, GPs scored higher than the general population whose mean scores were *M* = 2.83, *M* = 1.88 and *M* = 4.73 [23].

The findings from this study are in line with previous research. This study demonstrates that resilience in GPs is associated with psychological well-being, as has been found in other healthcare professionals [4–6]. Results also found that resilience was positively correlated with psychosocial work environment, which adds to the findings by Munn [24], stating that poor psychosocial work environment was associated with lower levels of resilience. The results confirm that interpretation bias is a key cognitive mechanism associated with resilience in GPs, in keeping with women living beyond cancer [9]. There has been little research on whether resilience is linked to emotion regulation [13,25]. Yet, this study also suggested that cognitive reappraisal (one form of emotion regulation) may be associated with being resilient as a GP, although it may be less significant when interpretation bias is considered.

A key strength of this study was that it used an experimental task (SST) to assess interpretation bias that is not subject to demand effects. Furthermore, our study presented a reasonable sample of GPs (*N* = 114) who work in a stressful occupational, at a time when COVID-19 was still impacting on their work and working environment. Another strength is that GPs from both urban and rural areas participated in the study. This is beneficial because it includes GPs from varying areas and our sample is representative of the UK GP population, though it must be noted that more participants were working in urban locations.

One study limitation is that the sample is not an even split of male and females, with 99 female GPs (91%) participating in the study, whilst only 9 (8%) males participated. The higher proportion of female to male GPs in the study does not match the actual proportion of male and female GPs in the UK. Another weakness may be that more resilient GPs were more likely to respond to the survey, as the more stressed and less resilient GPs may not have volunteered due to stress. Therefore, our sample may not include the least resilient GPs. The internal reliability of the SST in the present sample is also a limitation. The shortened SST had moderate internal reliability in our sample, and lower when adopting the split-half method. One reason could be that worries vary between individuals. For high internal consistency, most people would have to unscramble sentences exclusively positively or negatively, but this may not capture the nature of worries in our sample. Some items, such as ‘finding a job is easy/hard’, may be common worries for GPs, and thus even the GPs leaning strongly towards positive interpretation bias might unscramble this item in a negative way. There may also be other items that are a particular worry for specific participants who otherwise have made mainly positive interpretations. Nonetheless, our finding is at odds with a recent systematic review with meta-analysis of the SST which suggested the SST to have good convergent validity and consistency [26]. It may indicate that the 10-item worry version is less reliable than other SST versions, such as the 20-item worry and depression-related SST [22].

With regards to the internal and external validity of the findings, the authors acknowledge that this may present some limitations, particularly given the flexible portfolio of GP work. Whilst in our sample, participants worked an average of 5.5 GP sessions per week and thus were providing regular and direct patient contact, there may nonetheless be some variation in the amounts and ways of working across GPs sampled. However, it should be noted that all GPs had the same basic training requirements and worked to the same set of professional standards, as outlined by the General Medical Council (GMC). To this extent, the authors err on the side of caution in generalizing the findings of this study to the wider GP population and do not claim the sample to be fully representative. The authors also recognize the working model of GPs is different from medical doctors working within UK hospital-based settings, meaning results should be interpreted with caution, and in context.

Future research could focus on a larger, longitudinal study, as it would be beneficial to replicate the present results and assess whether these cognitive mechanisms predict future resilience of GPs. It would be interesting to replicate the study with other highly stressful medical occupations, such as with nurses, paramedics and secondary care doctors, to investigate if there are similar correlations between the cognitive mechanisms and resilience.

The main implication from this study is that positive interpretation bias is associated with resilience and could have a key role in maintaining resilience. Furthermore, this mechanism could form a target for interventions designed to promote resilience. Hirsch and colleagues [11] promoted more positive interpretations in people with generalized anxiety and this reduced anxiety and depression in the longer term [11]. Given that interpretation bias is associated with resilience, training more positive interpretations could promote resilience in GPs with lower levels of resilience, in addition to supporting wider policy and practice changes in the profession.
